# Giant Paraganglioma Complicated With Catecholamine Crisis and Catecholamine Cardiomyopathy: A Case Report and Review of the Literature

**DOI:** 10.3389/fendo.2021.790080

**Published:** 2022-02-03

**Authors:** Xiangping Ma, Zhen Chen, Peng Xia, Chunmei Zhang, Keqiang Yan, Yidong Fan, Yingli Wang, Yun Ti, Peili Bu

**Affiliations:** ^1^ Key Laboratory of Cardiovascular Remodeling and Function Research, Chinese Ministry of Education, Chinese National Health Commission and Chinese Academy of Medical Sciences, State and Shandong Province Joint Key Laboratory of Translational Cardiovascular Medicine, Department of Cardiology, Qilu Hospital, Cheeloo College of Medicine, Shandong University, Jinan, China; ^2^ Department of Cardiology, Liaocheng People’s Hospital, Liaocheng Clinical School of Shandong First Medical University, Liaocheng, China; ^3^ Department of Urology, Qilu Hospital of Shandong University, Jinan, China

**Keywords:** giant paraganglioma, catecholamine crisis, catecholamine cardiomyopathy, heart failure, case report

## Abstract

**Background:**

Pheochromocytomas and paragangliomas (PPGL) are rare neuroendocrine tumors which overproduce catecholamines. Heart failure and myocardial infarction caused by paraganglioma complicated with catecholamine crisis are the most common causes of death in PPGL patients before surgery. When giant paraganglioma is complicated with catecholamine crisis, treatment brooks no delay.

**Case Summary:**

A 49-year-old man had episodic sweating, tachycardia with irregular pulse, and headaches 5 days before, and then showed up with chest pain and wheezing for 1 day. Meanwhile, he developed symptoms of recurrent severe abdominal pain and loss of consciousness, and his blood pressure was severely unstable (from 70/40 to 300/200 mmHg). First, the electrocardiogram showed ventricular tachycardia, and then we noticed the waves of ST-segment elevation, but we did not find significant abnormalities in coronary angiography. Abdominal CT and MRI revealed a giant lesion with bleeding or infection in the retroperitoneal adrenal area. These imaging findings were confirmed during surgery, and there was vascular adhesion between the retroperitoneal tumor and the inferior vena cava and left and right renal vein. After the successful resection of the tumor, postoperative pathology confirmed paraganglioma, and the patient pulled through and was discharged quickly.

**Discussion:**

This is a rare case of giant paraganglioma complicated with catecholamine crisis and catecholamine cardiomyopathy. We can diagnosis this disease greatly by elevated norepinephrine, and it is a gold biochemical standard at present. Standard treatment is surgical resection, which is effective in treating this rare neuroendocrine tumor.

## Introduction

Paragangliomas are unusual neuroendocrine tumors, and they originate from chromaffin cells of the parasympathetic or sympathetic paraganglia outside of the adrenal glands. Clinical symptoms include hypertension, metabolic disorders, and others. The intermittent nature of these symptoms often leads to a delay in the diagnosis. Patients rarely initially present with cardiac complications such as myocardial infarction, heart failure, or arrhythmias. Chest pain or distress is not quite common, which is only seen in approximately 18% of the patients (1, 2). Paraganglioma-associated cardiomyopathy is due to adrenergic cardiac toxicity. Giant paraganglioma complicated with catecholamine crisis is a rare and dreaded complication.

In this article, we showed an uncommon and critical case of a giant paraganglioma complicated with catecholamine crisis. The patient presented with symptoms of myocardial infarction and heart failure. The optimal treatment is surgery to remove the lesion and thereby improve survival as well as restore cardiac function (3).

## Case Presentation

Pheochromocytoma and paraganglioma, first completely described by Felix Fraenkel (4), are rare neuroendocrine tumors. A 49-year-old man was transferred to the cardiac care unit of Qilu Hospital of Shandong University from Liaocheng People’s Hospital, who presented with a 5-day history of episodic sweating, tachycardia with irregular pulse, and headaches and then a 1-day history of chest pain and wheezing. Meanwhile, there was no obvious cause of recurrent severe abdominal pain and loss of consciousness. During the period of hospitalization, his blood pressure was severely unstable. The maximum blood pressure was 300/200 mmHg, and the minimum blood pressure was 70/40 mmHg. There were no other diseases except for varicose veins of the lower extremities surgically treated 10 years ago, and he used to be healthy. He had no allergies, no family history of heart disease, and no history of smoking or drinking. On presentation, his heart rate was 104 beats/min and his blood pressure was 200/170 mmHg. On examination, the abdomen was soft and the mass was barely palpable; there was no regional lymphadenopathy, pain, or discomfort. The remainder of the examination, including heart and nervous system examination, was normal.

Laboratory tests revealed a significant increase in catecholamine series, including norepinephrine (193.74 nmol/L, normal range 0~5.17 nmol/L), epinephrine (7.18 nmol/L, normal range 0~0.34 nmol/L), normetanephrine (35.46 nmol/L, normal range 0~0.71 nmol/L), metanephrine (2.5 nmol/L, normal range 0~0.42 nmol/L), and vanillylmandelic acid (378.49 nmol/L, normal range 0~62 nmol/L). The levels of biomedical marker of myocardial injury were also increased, cTnI (0.10 ng/ml, normal range 0.01–0.023 ng/ml), and NT-pro-BNP was 3,970 pg/ml (<125 pg/ml can exclude heart failure), and the highest was 8,081 pg/ml. The inflammatory index was higher than normal, such as the level of hypersensitive C-reactive protein (127.54 ng/L, normal range 0–10 ng/L), PCT (0.432 mg/L, normal range <0.1 ng/ml), white blood cell count [14.15 × 10^9^/L, normal range (4~10) × 10^9^/L], and neutrophil ratio (88.70%, normal range 40%–75%). Liver function was impaired (ALT 171 U/L, AST 227 U/L, normal range <45 U/L). Renal function, cortisol, renin, thyroxine, angiotensin, and aldosterone were not affected.

**Figure 1:**
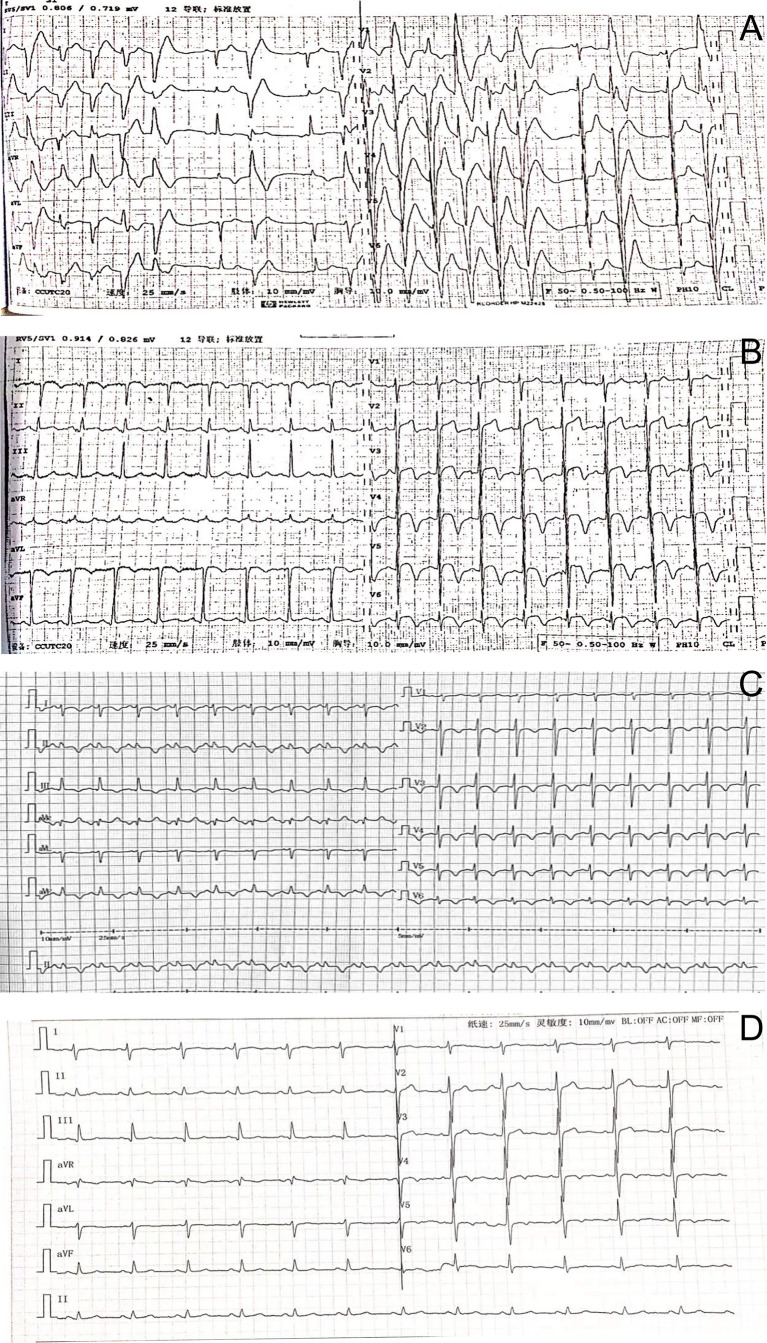
**(A)** The electrocardiogram showed ventricular tachycardia. **(B)** Electrocardiography on admission showing the waves of ST-segment elevation in leads I, II, aVF, and V2–V6. **(C)** Electrocardiography showed inverted T waves in leads I, II, III, aVF, and V2~6, and a prolonged QT interval. **(D)** Three months after surgery, T waves having slight changes were revealed.

**Figure 2:**
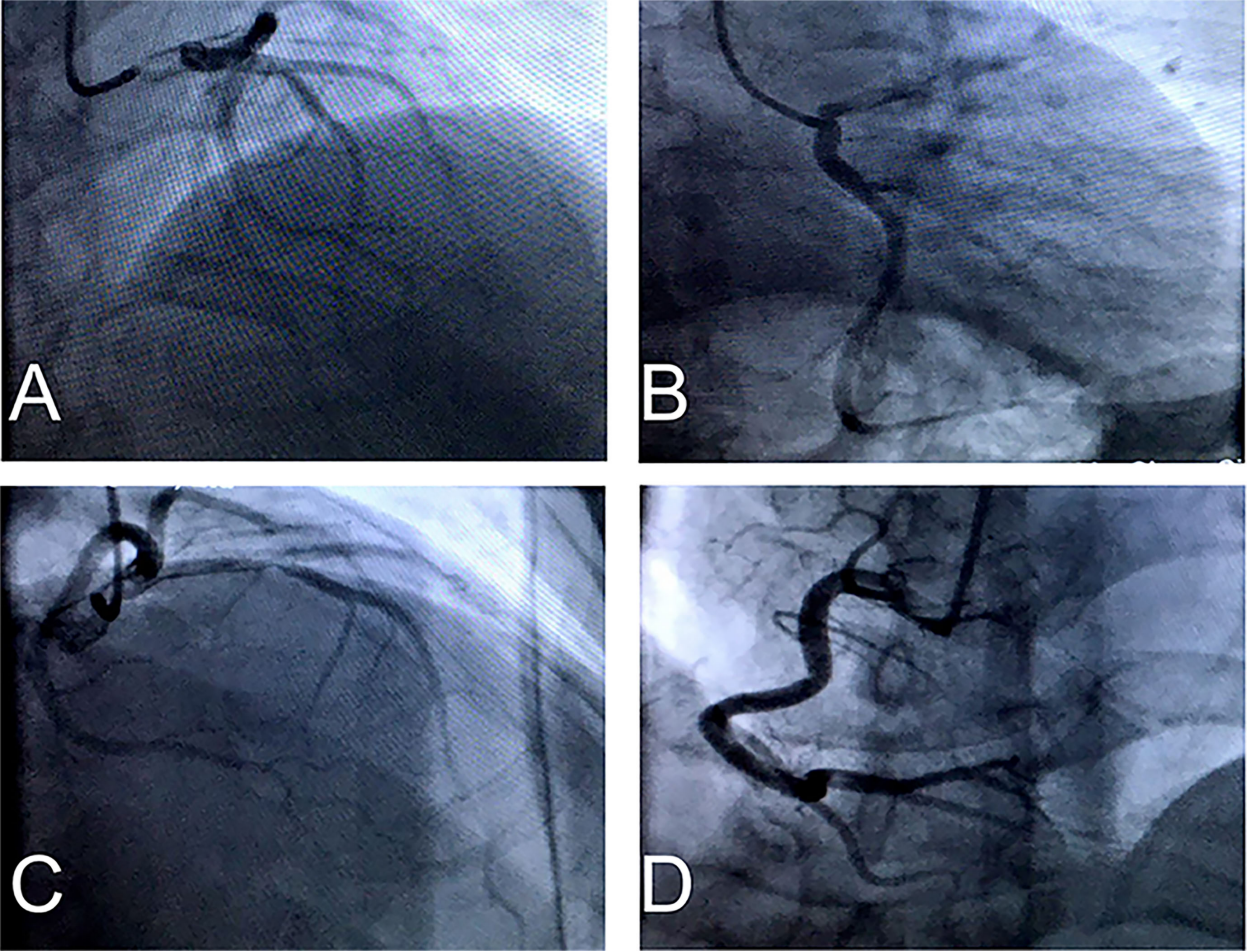
**(A–C)** Emergency coronary angiography showed LAD emitted D1 have plaque formation and mild stenosis, but LCX have no obvious stenosis. **(D)** RCA have no significant abnormality.

Electrocardiogram showed ventricular tachycardia (**Figure 1A**) and the waves of ST-segment elevation in leads I, II, aVF, and V2–V6 in the acute phase (**Figure 1B**), followed by diffuse inverted T waves in leads I, II, III, aVF, and V2~V6 (**Figure 1C**). TTE showed that four cardiac chambers were not affected, the interventricular septum was slightly thicker (13 mm, normal range 8~11 mm), and the ejection fraction was 37%. The middle and lower segment of the interventricular septum, the middle and lower segment of the left ventricular lateral wall and inferior wall, and the lower segment of the anterior wall and left ventricular apex almost disappeared. Emergency coronary angiography (**Figure 2**) did not show significant abnormalities. It only showed that LAD emitted D1 with plaque formation and mild stenosis.

**Figure 3:**
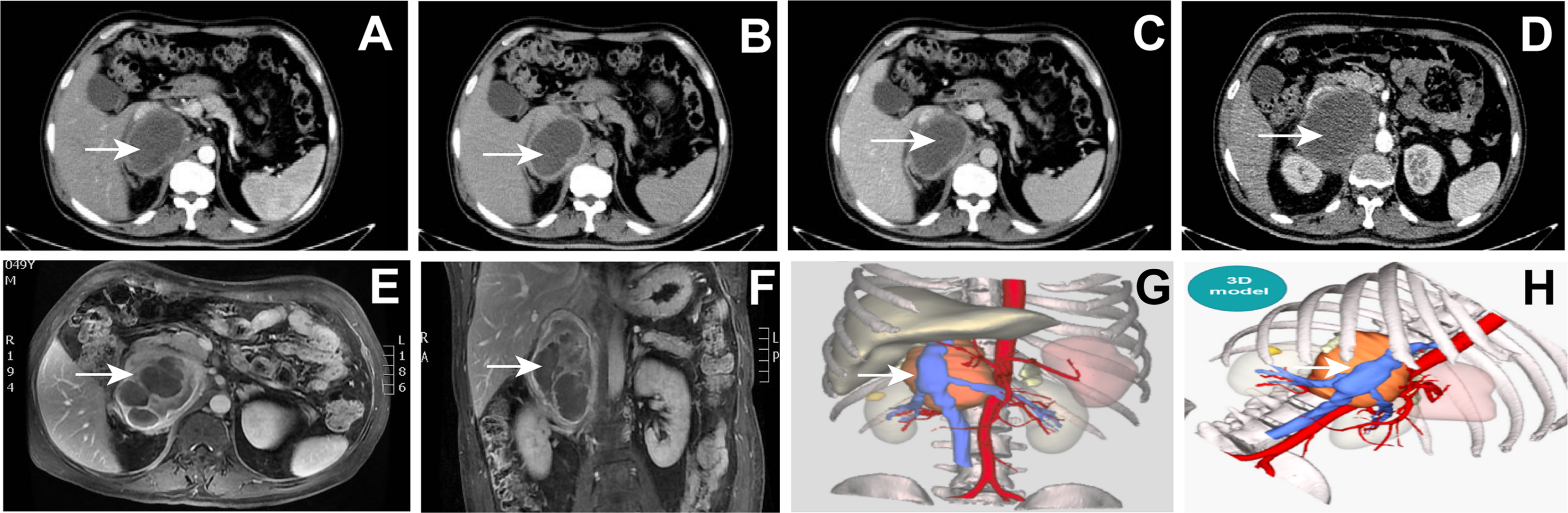
**(A–D)** Abdominal plain CT: right retroperitoneal cystic solid space-occupying lesion (arrow). **(E–F)** Magnetic resonance image of the abdomen with gadolinium contrast showing a 9.0* 6.5 * 10.7 cm right adrenal mass (arrow). **(G, H)** The 3D model of the abdominal lesion (arrow).

Despite the symptoms of headaches, no obvious abnormality was found in plain brain scan CT. Abdominal plain CT showed that a soft tissue density shadow was seen in the right adrenal region (the size was about 9.3 * 6.7 cm, the density was uneven, and the inferior vena cava was pressed). Enhancement after abdominal plain scan and the 3D models showed the following (**Figures 3A**–**D, G, H**): there was a right retroperitoneal cystic solid space-occupying lesion, and it was complicated with bleeding or infection; adjacent to the retroperitoneal space, there were a small amount of exudative changes around the inferior vena cava and also fascia thickening. Magnetic resonance imaging (MRI) of the abdomen with gadolinium contrast showed the following (**Figures 3E, F**): there was a right retroperitoneal space-occupying lesion, the tumor with bleeding or infection was considered, and the tumor size was about 9.0 * 6.5 * 10.7 cm.

Based on the relevant examination results, we discussed his condition in the department. At first, he presented symptoms of episodic sweating and tachycardia. Then, his blood pressure was severely unstable and his electrocardiogram showed ventricular tachycardia, ST-segment elevation, and diffuse inverted T waves. Therefore, we considered differential diagnoses including thyroid crisis and acute coronary syndrome. However, his thyroxine was in the normal range, and coronary angiography did not show significant abnormalities. Combined with his obvious symptoms of heart failure and the radiographic results, we considered that he was likely to be diagnosed with phaeochromocytomas and paragangliomas (PPGL) because PPGL could secrete a lot of catecholamine, including norepinephrine and epinephrine, which resulted in his blood pressure being severely unstable and his cardiovascular system being destroyed.

**Figure 4:**
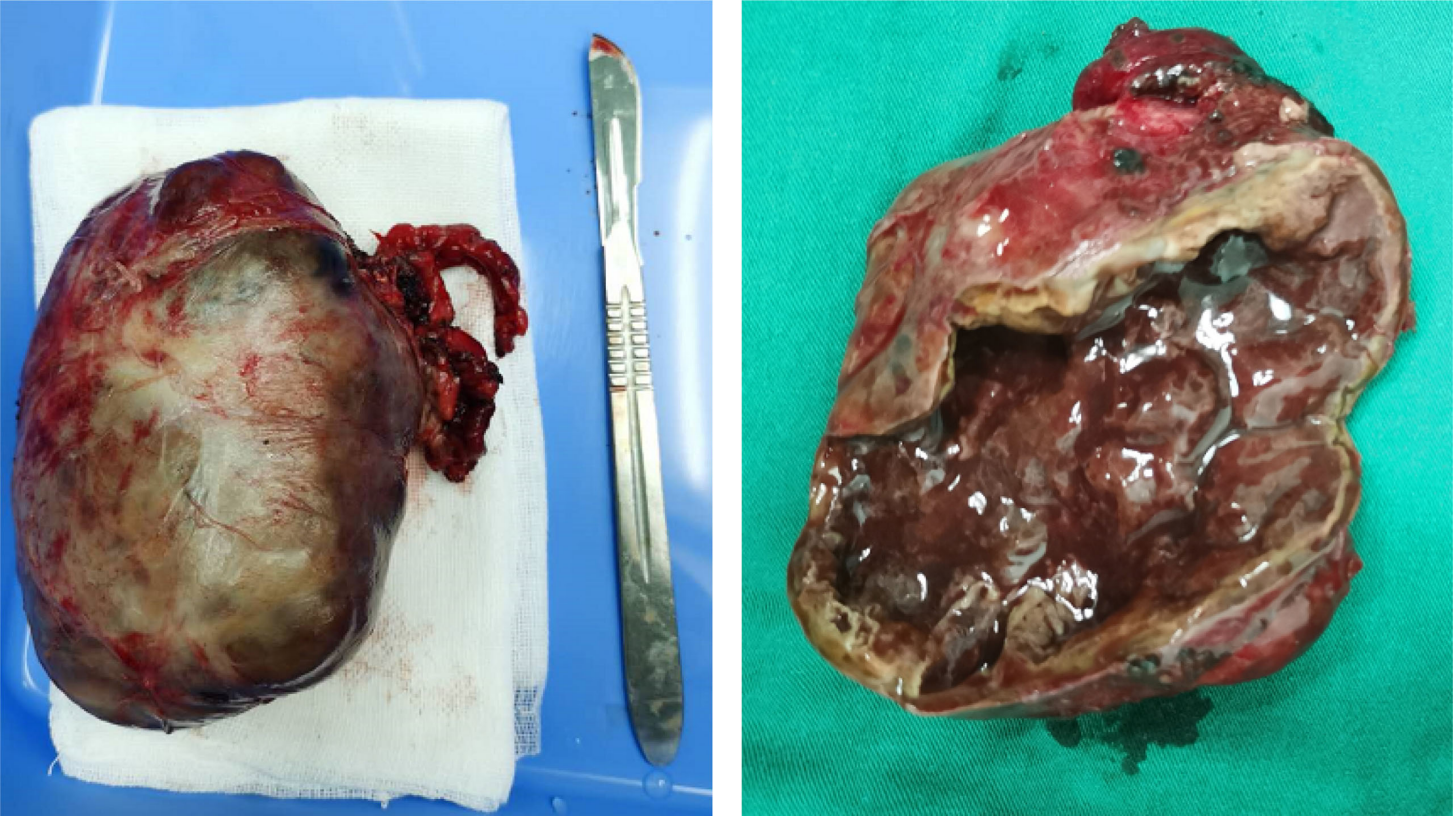
Macroscopic examination of the tumor (9 * 8 cm) showed areas of necrosis and hemorrhage of the right retroperitoneal.

According to his relevant examination results and symptoms, we recommended that the patient should undergo surgery to remove the tumor. In order to get better operation result, preoperative preparation was very important and professional. First, we monitored his blood pressure every day and actively controlled it. He took phenoxybenzamine to control his blood pressure at first, but his blood pressure was still unstable. So, we added beta (β)-adrenoceptor blockers, and his blood pressure was controlled very well. Then, we administered amiodarone and esmolol to prevent and treat paroxysmal ventricular tachycardia. At the same time, we gave standardized treatments for his heart failure. After a period of systematic treatments, his blood pressure was stable, his electrocardiogram did not show ventricular tachycardia again, and his symptoms of heart failure were markedly improved. Then, the patient was transferred to the urology ward, where resection of the right retroperitoneal tumor under general anesthesia was performed. During surgery, it was seen that the postcava was lifted and thinned, and we carefully separated the vascular adhesion between the retroperitoneal tumor and the inferior vena cava and left and right renal vein and turned the liver to the left to expose the retroperitoneal tumor. The tumor was located in front of the right adrenal gland and it clearly oppressed the adrenal gland. The tumor was carefully separated from the surrounding tissue, and it was removed (**Figure 4**). The size of the tumor was about 9 * 8 cm, and it was cystic and solid and sent for pathological examination. The patient was transferred to the urology ward after the surgery.

The patient had an uneventful course and was discharged 12 days after surgery. After discharge, β-adrenoceptor blockers (metoprolol) were given to the patient to stabilize his condition.

**Figure 5:**
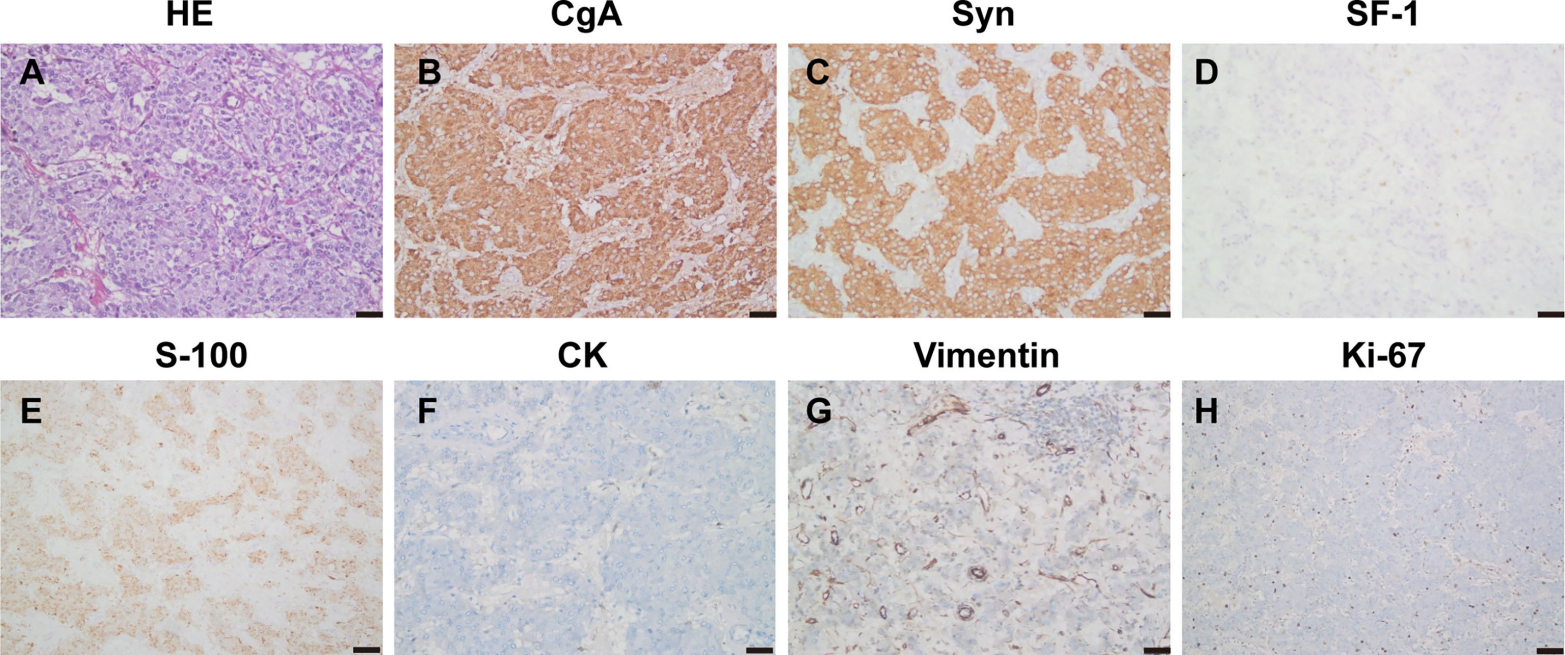
Pathology and immunohistochemistry **(A–H)** showed HE staining of the tumor tissue (HE, ×200), CgA (+), Syn (+), SF-1 (−), S-100 (+), CK (−), and vimentin (−), and the Ki-67 index was 2%.

Histopathology of the resected specimen confirmed the diagnosis (**Figure 5**). The specimen (retroperitoneal) showed that most of the tumors were hemorrhagic and necrotic, and only a few tumor tissues remained. Combined with morphology, immunohistochemistry, and clinical description, the tumor was considered as paraganglioma with a volume of about 10 * 8 * 4 cm. Immunohistochemistry showed CgA (+), Syn (+), SF-1 (−), S-100 (+), Melan-A (−), HMB45 (−), CK (−), and vimentin (−), and the Ki-67 index was 2%. The patient received genetic testing and no pathogenic variants were found.

## Follow-Up

We followed up the patient 3 months later, and he was free of episodic sweating, tachycardia, or headaches, without evidence of recurrence. Moreover, his electrocardiogram (**Figure 1D**) showed that T wave changes slightly as confirmed by an outpatient follow-up.

One year after the operation, we conducted a systematic follow-up of the patient again. Laboratory tests showed that the catecholamine series were sustained in the normal range, including norepinephrine (1.53 nmol/L, normal range 0~5.17 nmol/L), normetanephrine (0.69 nmol/L, normal range 0~0.71 nmol/L), and metanephrine (0.14 nmol/L, normal range 0~0.42 nmol/L). Meanwhile, adrenal enhanced CT was performed and it did not find new lesions. After questioning, he said that he had no obvious symptoms and he could work and live normally. According to his situation, we recommended that he return to the hospital for follow-up visit on time.

## Discussion

PPGL are uncommon tumors, and they originated from cells derived from the neural crest. The incidence of pheochromocytoma and paraganglioma is about 0.6 cases per 100,000 person-years (5, 6). The classical triad symptom includes tachycardia, headaches, and sweats. The intermittent nature of these symptoms often leads to a delay in the diagnosis. Patients rarely initially present myocardial infarction, heart failure, or arrhythmias. However, chest pain or distress is not quite common. Surgical resection is the mainstay treatment, which can improve cardiac function. Clinical manifestations include different cardiovascular signs and symptoms, which are related to excessive secretion of catecholamines (2, 3). Catecholamine crisis is a rare but dreaded complication (7). Patients with pheochromocytoma often have persistent or paroxysmal hypertension. Because catecholamines are rapidly and significantly released in the tumors, which endanger the function of vital organs, the blood pressure of some patients may reach dangerously high values above 200 mmHg. However, normal blood pressure or even hypotension is also common in patients with dopamine-producing paragangliomas, and there is also abrupt cessation of catecholamine secretion due to tumor necrosis, desensitization of adrenergic receptors, or hypocalcemia (8). These tumors are usually large, and the macroscopic examination of the tumor of the patient measuring 9 * 8 cm showed areas of necrosis and hemorrhage of the right retroperitoneal (**Figure 3**). PPGL can present with severe abdominal pain due to underlying intralesional bleeding (9).

Whenever patients with PPGL have heart failure and multisystem crisis symptoms without coronary heart disease, valvular heart disease, or unknown etiology, paraganglioma with catecholamine crisis should always be suspected. Paraganglioma complicated with catecholamine crisis is an acute reversible heart failure syndrome (10). The mechanisms of catecholamine-induced cardiomyopathy (CMP) include desensitization of β1-adrenoceptors, intracellular calcium overload, oxidative stress, and mitochondrial dysfunction (7). In our patient, due to abnormal electrocardiogram and elevated myocardial enzymes, cardioangiography was initially performed, and myocardial infarction was suspected. However, coronary angiography did not show significant abnormalities. This may result from catecholamine-induced reversible coronary vasoconstriction leading to myocardial ischemia (11). Paraganglioma may lead to life-threatening heart dysfunction. When giant paraganglioma is complicated with catecholamine crisis, treatment brooks no delay.

The reliable biochemical method for PPGL includes laboratory tests of plasma or urine. Presently, it is the gold biochemical standard to properly assess the elevation of norepinephrine. Plasma-free metanephrines or urinary fractionated metanephrines have the highest sensitivity for the diagnosis of pheochromocytoma. For tumors that secrete norepinephrine, radical operative treatment after appropriate drug therapy is the main treatment strategy. Ideally, tumor localization can only begin if there is clear biochemical evidence for phaeochromocytoma. The 2014 Endocrine Society clinical practice guidelines for pheochromocytoma and paraganglioma recommend CT rather than MRI as first-choice imaging modality to diagnose PPGL (5). Today, the most common method used for the initial localization of abdominal pheochromocytoma is CT scans of the entire abdomen.

Myocardial changes of most patients with giant paraganglioma complicated with catecholamine crisis can be improved after appropriate treatments. Combined alpha (α)- and beta (β)-adrenergic blockade is the standard treatment for patients with pheochromocytoma in order to prevent intraoperative hypertensive crises (1). Time for improvement of heart changes due to giant paraganglioma complicated with catecholamine crisis may take as short as 1–2 weeks or may be up to several months (5). Catecholamine excess in patients with PPGL can lead not only to LV hypertrophy, which is reversible following curative surgery intervention, but also to impairment of systolic LV function and subclinical alterations of diastolic LV function (12, 13). For this patient, we can pay attention to the follow-up. The follow-up of these patients is essential and should be done for an unlimited period of time (6). Diabetes is frequent in patients with pheochromocytoma and is related to catecholamine-induced insulin resistance or insulin suppression (14). However, in most cases, blood sugar can recover after the operation.

The patients should undergo genetic testing. In this test, mutations in genes encoding succinate dehydrogenase enzyme subunits are the most important genetic cause of these tumors (15). In our case, genetic testing was negative. About 10% of PPGL are metastatic, and they usually relate to germline mutations in the gene encoding succinate dehydrogenase complex iron sulfur subunit B (SDHB). As most PPGL are benign, surgical resection is the optimal method, and the recovery and prognosis are quite good after complete tumor resection (16).

In this case, the patient presented symptoms of episodic sweating, tachycardia, and headaches, and then his blood pressure was severely unstable. Therefore, we considered that he was likely to be diagnosed with PPGL, and the result of catecholamine series supported this diagnosis. Meanwhile, he had chest pain and his electrocardiogram showed ventricular tachycardia, ST-segment elevation, and diffuse inverted T waves. Additionally, laboratory tests, TTE, and the obvious symptoms of heart failure also suggested impairment of his cardiac function. To further clarify the diagnosis and the location of the lesion, CT and MRI were performed. According to the CT and MRI results, we recommended that the patient should undergo surgery to remove the tumor. After sufficient preoperative preparation, his blood pressure was stable, his electrocardiogram did not show ventricular tachycardia again, and his symptoms of heart failure were markedly improved. Then, he finished the surgery in the urology ward. As a result, his hemodynamic status was stable and he recovered quickly. After that, we followed up the patient several times, and he said that he had no obvious symptoms and he could work and live normally. In addition, his adrenal enhanced CT did not show new lesions.

## Conclusion

We reported a rare case of giant paraganglioma complicated with catecholamine crisis and catecholamine cardiomyopathy. We can diagnosis this disease greatly by elevated norepinephrine, and it is a gold biochemical standard now. The effective treatment of the disease related both to PHEO and PGL is tumor resection, and it is the sole therapy to cure this rare neuroendocrine tumor probably. Because of the metastatic potential of PPGL, it is meaningful to arrange a clinical follow-up for a long time.

## Data Availability Statement

The original contributions presented in the study are included in the article/supplementary material. Further inquiries can be directed to the corresponding author.

## Ethics Statement

The studies involving human participants were reviewed and approved by the Ethics Committee of Qilu Hospital of Shandong University. The patients/participants provided their written informed consent to participate in this study. Written informed consent was obtained from the individual(s) for the publication of any potentially identifiable images or data included in this article.

## Author Contributions

XM, ZC, PX, and CZ wrote the report. KY and YF took the pictures. YT, YW, and PB performed the research and revised the report. All authors contributed to the article and approved the submitted version.

## Funding

This work was supported by the State Key Program of the National Natural Science Foundation of China (grant number 81530014), National Key R&D Plan of China (grant number 2017YFC1700502), National Natural Science Foundation for Young Scientists of China (grant number 81700366), Natural Science Foundation of Shandong Province (ZR2019QH010), Cardiovascular Multidisciplinary Integrated Research Fund (z-2016-23-2101-10), and the Program of Educational Reform and Research Project of Cheeloo College of Medicine, Shandong University (qlyxjy-202037).

## Conflict of Interest

The authors declare that the research was conducted in the absence of any commercial or financial relationships that could be construed as a potential conflict of interest.

## Publisher’s Note

All claims expressed in this article are solely those of the authors and do not necessarily represent those of their affiliated organizations, or those of the publisher, the editors and the reviewers. Any product that may be evaluated in this article, or claim that may be made by its manufacturer, is not guaranteed or endorsed by the publisher.

## Abbreviations

PPGL, pheochromocytoma and paraganglioma; MRI, magnetic resonance image; TTE, transthoracic echocardiography; NT-pro-BNP, N-terminal pro-brain natriuretic peptide; cTnI, cardiac troponin I; ALT, alanine aminotransferase; AST, aspartate aminotransferase; MI, myocardial infarction; LAD, left anterior descending artery; PHEO, pheochromocytoma; PGL, paraganglioma; CMP, catecholamine-induced cardiomyopathy; LV, left ventricle; SDHB, succinate dehydrogenase complex iron sulfur subunit B.
